# Approach for the Design of Covalent Protein Kinase Inhibitors via Focused Deep Generative Modeling

**DOI:** 10.3390/molecules27020570

**Published:** 2022-01-17

**Authors:** Atsushi Yoshimori, Filip Miljković, Jürgen Bajorath

**Affiliations:** 1 Institute for Theoretical Medicine Inc., 26-1, Muraoka-Higashi 2-Chome, Fujisawa 251-0012, Japan; yoshimori@itmol.com; 2 Department of Life Science Informatics and Data Science, B-IT, LIMES Program Unit Chemical Biology and Medicinal Chemistry, Rheinische Friedrich-Wilhelms-Universität, Friedrich-Hirzebruch-Allee 6, D-53115 Bonn, Germany; miljkovi@bit.uni-bonn.de

**Keywords:** deep machine learning, generative modeling, kinase inhibitor design, Bruton’s tyrosine kinase, covalent inhibitors

## Abstract

Deep machine learning is expanding the conceptual framework and capacity of computational compound design, enabling new applications through generative modeling. We have explored the systematic design of covalent protein kinase inhibitors by learning from kinome-relevant chemical space, followed by focusing on an exemplary kinase of interest. Covalent inhibitors experience a renaissance in drug discovery, especially for targeting protein kinases. However, computational design of this class of inhibitors has thus far only been little investigated. To this end, we have devised a computational approach combining fragment-based design and deep generative modeling augmented by three-dimensional pharmacophore screening. This approach is thought to be particularly relevant for medicinal chemistry applications because it combines knowledge-based elements with deep learning and is chemically intuitive. As an exemplary application, we report for Bruton’s tyrosine kinase (BTK), a major drug target for the treatment of inflammatory diseases and leukemia, the generation of novel candidate inhibitors with a specific chemically reactive group for covalent modification, requiring only little target-specific compound information to guide the design efforts. Newly generated compounds include known inhibitors and characteristic substructures and many novel candidates, thus lending credence to the computational approach, which is readily applicable to other targets.

## 1. Introduction

Increasing interest in artificial intelligence methods is impacting computer-aided drug design and widening its scope [1]. Generative modeling is among the new approaches enabled through the application of deep neural network architectures [1,2,3,4]. It aims to produce novel chemical entities through deep learning from existing chemical matter, either by generally expanding biologically relevant chemical space through the generation of novel virtual libraries or by focusing on compounds with specific biological activities [2,3,4]. Although generative modeling is intensely investigated at present, reports of practical applications impacting medicinal chemistry are still rare [1]. This is typically the case for newly introduced (computational and experimental) methodologies, which will require time until they mature and measurably contribute to the practical drug design and medicinal chemistry programs.

So far, most drug design efforts have concentrated on generating reversible non-covalent inhibitors of target proteins, a hallmark of small-molecule drug discovery. In contrast, covalent inhibitors have experienced comparatively little interest, especially in the era of molecular and structure-based approaches [5]. Most covalent inhibitors permanently disable biological targets and ultimately lead to their degradation. Hence, covalent inhibitors are often associated with unfavorable pharmacological properties and undesired side effects, due to the non-selective inhibition of targets. However, these views have partly changed over the past decade as potential advantages of covalent inhibitors have increasingly been realized if unique or only weakly conserved residues important for the activity of given targets can be modified [5,6], leading to so-called targeted covalent inhibitors (TCIs) [6]. Often quoted favorable properties of TCIs include, among others, a high degree of target occupancy, long physiological half-life and ensuing high efficacy, or potential decoupling of pharmacodynamic and pharmacokinetic effects [5,6].

For the generation of reactive groups in TCIs that form covalent bonds to side-chain atoms of cysteine, lysine, or tyrosine residues, often termed chemical “warheads”, a variety of chemical reactions are applicable [6]. In addition, to facilitate non-permanent inhibition by TCIs, chemistry is also available to achieve covalent-reversible inhibition, which balances advantages of non-covalent as well as covalent interference with given targets [6].

Protein kinase inhibition is not only one of the major focal points of contemporary drug discovery efforts [7] but also a growth area for covalent inhibition. This is the case because most non-covalent kinase inhibitors developed thus far target the highly conserved ATP cofactor binding site in the catalytic kinase domain, giving rise to potential off-target promiscuity [7,8]. Accordingly, in kinase drug discovery, covalent inhibition is also considered as a mechanism to render the inhibitor selective for confined subsets of kinases having free cysteine residues in the active site region that are only little conserved across the human kinome [8].

**Figure 1:**
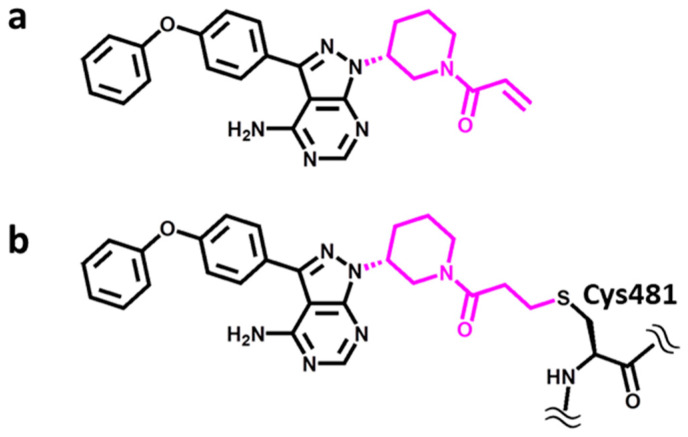
Structure of ibrutinib. The covalent drug ibrutinib contains an acrylamide warhead, colored magenta in (**a**), which forms a covalent bond to the thiol group of a Cys481 in BTK, shown in (**b**).

A representative and instructive example is provided by Bruton’s tyrosine kinase (BTK) [9], which belongs to the TEC (gene) family of non-receptor tyrosine kinases [10]. TEC kinases are expressed in hematopoietic, kidney, and liver cells and implicated in T-helper-cell activation through participation in cytokine receptor-dependent signaling pathways [10]. Hence, this kinase family includes therapeutic targets for the treatment of inflammatory diseases and leukemia, with BTK being the most intensely studied member and a major drug target [11]. For BTK, a variety of non-covalent as well as covalent inhibitors have been reported over the years [12]. Importantly, BTK is a primary target of the marketed covalent drug ibrutinib [13], depicted in Figure 1. Ibrutinib contains an acrylamide warhead acting as a Michael acceptor in the formation of a covalent bond with the thiol group of a cysteine residue in the active site of BTK (Cys481).

In this work, we have addressed the question of whether novel covalent inhibitors of BTK could be designed via deep generative modeling by focusing on ibrutinib as a template and its interactions with BTK. To our knowledge, herein, we introduce the first generative design strategy for covalent enzyme inhibitors and report a number of new BTK candidate compounds for follow-up investigations in medicinal chemistry.

## 2. Results and Discussion

### 2.1. Selected Covalent BTK Inhibitors

We aimed to design covalent BTK inhibitors containing an acrylamide/Michael acceptor warhead, for which the drug ibrutinib served as a template [13], as shown in Figure 1a. The warhead reacts with the SH-group of cysteine residues, forming a covalent bond (Figure 1b). Hence, kinases having a free cysteine within or in the vicinity of the active site (including the cofactor and substrate binding site) might be inhibited by such compounds. However, this is only possible if the warhead can reach the thiol group of cysteine residues and be accommodated in the binding site, which might be prevented, for example, by steric hindrance or other chemical incompatibilities. This offers opportunities to render covalent inhibitors target-selective by modifying the remaining non-reactive parts of their structure to fit into a given binding site. In any event, this specific mode of covalent inhibition principally limits potential kinase targets to a subset of kinases having a free cysteine in the active site region. BTK contains a free cysteine in the F2 subsite (αD-1 position) in the front region of the ATP cofactor binding site, the location of which is shared by a total of 12 human kinases (plus isoforms) [8].

### 2.2. Inhibitor Distribution

We searched ChEMBL [14] for covalent BTK inhibitors containing the piperidine-based Michael acceptor warhead of ibrutinib, for which high-confidence activity data were available, and identified a total of 34 such inhibitors, shown in Supplementary Figure S1. We then searched ChEMBL for covalent inhibitors of other kinases having the same warhead and identified such inhibitors for a total of 20 kinases, with 1–35 inhibitors per kinase, as reported in Table 1. These included several kinases with a cysteine at the position corresponding to BTK but also others with a free cysteine at a different position. Eighteen of the 20 kinases were found to share varying numbers of inhibitors with BTK. Among these was erbB1 with 35 inhibitors. For BTK and erbB1, most inhibitors belonging to this class were available, with 34 and 35 compounds, respectively. BTK and erbB1 have a free cysteine at corresponding positions in their structure, but only share one covalent inhibitor with the piperidine-based Michael acceptor warhead (Table 1), hence indicating the potential for selective covalent inhibition of related kinases. We also found that 7 of the 34 BTK inhibitors were promiscuous on the basis of high-confidence activity data, i.e., they were active against two or more kinases. Promiscuous inhibitors included ibrutinib, reported to be active against a total of 11 targets. The remaining 27 inhibitors were active against BTK. Data available for ibrutinib, which represents an extensively investigated drug, might provide a realistic estimate for the degree of selectivity that can be expected for this class of inhibitors, although other BTK inhibitors containing this warhead might be more selective than ibrutinib, given their steric and chemical features.

### 2.3. Artificial Intelligence-Assisted Inhibitor Design

New BTK candidate inhibitors were designed using the DeepSARM, which combines the SAR matrix (SARM) data structure with deep learning and generative modeling [15]. The methodology is detailed in the Supplementary Methods. The underlying principles are as follows: From a given compound dataset, the SARM approach extracts all structurally related analogue series and organizes these series in matrices reminiscent of R-group tables, as shown in Supplementary Figure S2a. This is facilitated by applying a dual-compound fragmentation scheme yielding core structure fragments (Keys) and substituents (Values). In the first round, compounds are fragmented, yielding a Key 1 and Value 1 fragment, and in the second round, the Key 1 fragments from the first fragmentation, yielding a Key 2 and Value 2 fragment. This fragmentation scheme identifies all compounds and core structures that are only distinguished by a chemical change at a single site. Accordingly, each qualifying Key 2 fragment represents a series of analogues with structural modifications at a single site and each SARM contains a subset of structurally closely related series with core fragments distinguished by a structural change at a given site. As such, cells in the SARM represent individual dataset compounds and empty cells represent currently unexplored combinations of Key 1 and Value 1 fragments, providing candidate compounds for series expansion (Supplementary Figure S2a).

**Figure 2:**
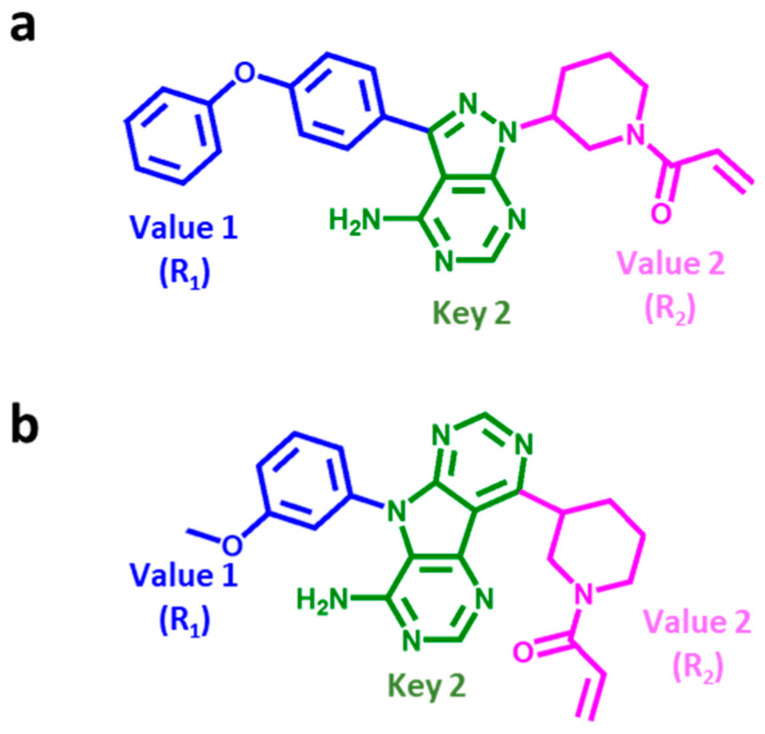
Key 2, Value 2, and Value 1 fragment assembly. Shown is the DeepSARM fragment composition of (**a**) ibrutinib and (**b**) a candidate compound. Key 2, Value 2 (R_2_), and Value 1 (R_1_) are displayed in green, magenta, and blue, respectively.

Based upon this hierarchical decomposition scheme and the ensuing SARM data structure, a compound design strategy can be implemented to explore combinations of novel fragments as follows: Combinations of Key 2 and Value 2 fragments yield Key 1, i.e., complete core structures, of novel compounds. If the resulting core structures are combined with newly generated Value 1 fragments, new compounds are obtained. For ibrutinib, the corresponding ([Key 2 − Value 2] − Value 1) fragment assembly is illustrated in Figure 2a and a candidate compound containing two novel (Key 2, Value 1) fragments in Figure 2b. Importantly, for the design of covalent BTK inhibitors attempted herein, Value 2 fragments completing the inhibitor core structure are required to contain the invariant warhead.

Following this general design approach, new Key 2, Value 2, and Value 1 fragments are required to obtain new compounds. The generation of Key 2, Value 2, and Value 1 is facilitated using DeepSARM. To further expand the close-in analogue design space provided by SARM, DeepSARM is composed of three sequence-to-sequence (Seq2Seq) models representing an encoder–decoder framework for learning the corresponding structural fragments and generating new ones. These Seq2Seq models represent a recurrent neural network architecture successfully used in natural language processing to transform a sequence of characters into another (hence the name). The Seq2Seq models in DeepSARM are also termed Key, Value 2, and Value 1 Generator, respectively. The DeepSARM architecture is illustrated in Supplementary Figure S2b. To expand the compound design space, DeepSARM is first pre-trained on a large set of compounds (for instance, a collection of kinase inhibitors across the human kinome) and then fine-tuned on a smaller compound set (such as known inhibitors of a specific kinase target). The generation of a SARM with compounds composed of new fragments from DeepSARM is illustrated in Supplementary Figure S2c.

DeepSARM fragment design via Seq2Seq models is guided by cumulative log-likelihood scores from the Seq2Seq models (see the Supplementary Methods). Given the derivation of this scoring function, small scores close to 0 are obtained for compounds whose structural fragments are similar to known inhibitors or identical and large scores approaching 1 for compounds with novel fragments not contained in the training data. Hence, increasing log-likelihood scores indicate the structural novelty of candidate compounds.

### 2.4. BTK Inhibitor Design

DeepSARM was pre-trained with 45,441 kinase inhibitors from the Kinase SARfari collection of ChEMBL [14] and then fine-tuned using the 34 covalent BTK inhibitors with the piperidine-based Michael acceptor warhead depicted in Supplementary Figure S1. Hence, only a small set of inhibitors was used for fine-tuning of the generative model.

#### 2.4.1. Key 2 Structures

**Figure 3:**
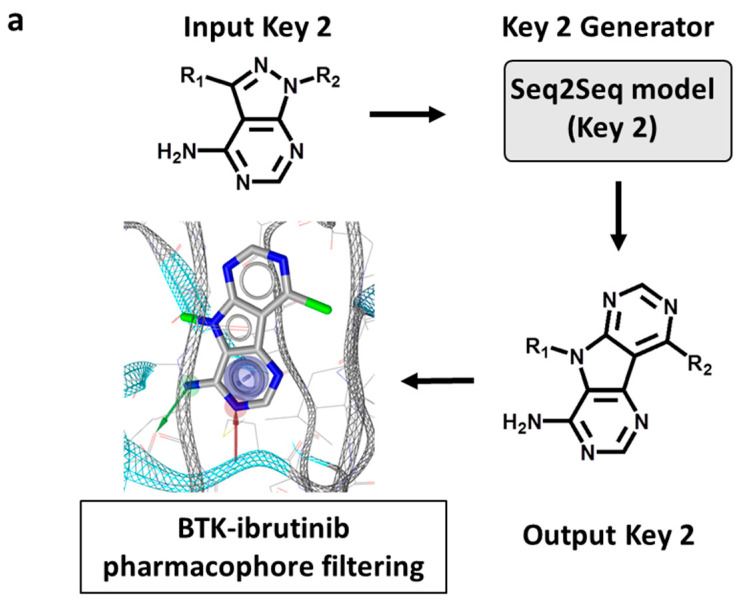

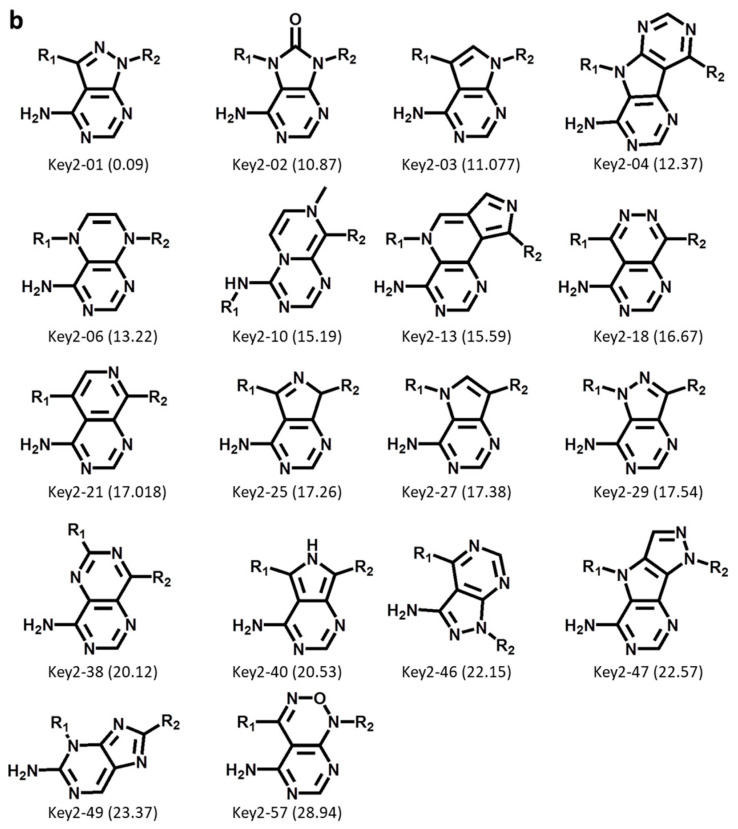
Generation of new Key 2 fragments. (**a**) The computational workflow for generating Key 2 fragments using the Seq2Seq model (Key 2) and pharmacophore filtering. In the lower left image, pharmacophore features including hydrogen bond donor, hydrogen bond acceptor, and aromatic groups are shown as a green arrow, red arrow, and blue circle, respectively. (**b**) The structure of 18 newly generated Key 2 fragments. Below each structure, the identification number and log-likelihood score (in parentheses) are provided.

First, Key 2 fragments were generated, representing the major substructure of the inhibitor scaffold, and evaluated using an ibrutinib core structure-based pharmacophore model based upon the ibrutinib-BTK X-ray complex structure (see Section 3), as illustrated in Figure 3a. Accordingly, from 50,000 initially sampled Key 2 structures, 59 Key 2 fragments passing the rotational bond filter and the pharmacophore filter were selected, and 18 of these fragments were prioritized that closely matched the pharmacophore, depicted in Figure 3b. These structures included a variety of modifications of the ibrutinib Key 2, including the introduction or replacement of ring heteroatoms and, interestingly, tricyclic Key 2 variants. These findings confirmed the ability of DeepSARM to generate a considerable spectrum of scaffold modifications compared to the original core structures of BTK inhibitors used for fine-tuning.

We then encoded only the cyclic structures of Key 2 fragments of newly designed BTK inhibitors for substructure searching in ChEMBL or only kinase and BTK inhibitors with available high-confidence activity data. As reported in Table 2, 6 of the 18 Key 2 fragments were not detected in ChEMBL. Moreover, 8 and 14 Key 2 fragments were novel in all kinase inhibitors or only BTK inhibitors, respectively, while the remaining structures were already available. These findings confirmed the ability of DeepSARM to regenerate known inhibitory structural motifs and generate novel structures, hence providing a variety of plausible hinge-binding motifs for covalent BTK inhibitors and lending further credence to the design approach.

#### 2.4.2. Value 2 and Value 1 Structures

**Figure 4:**
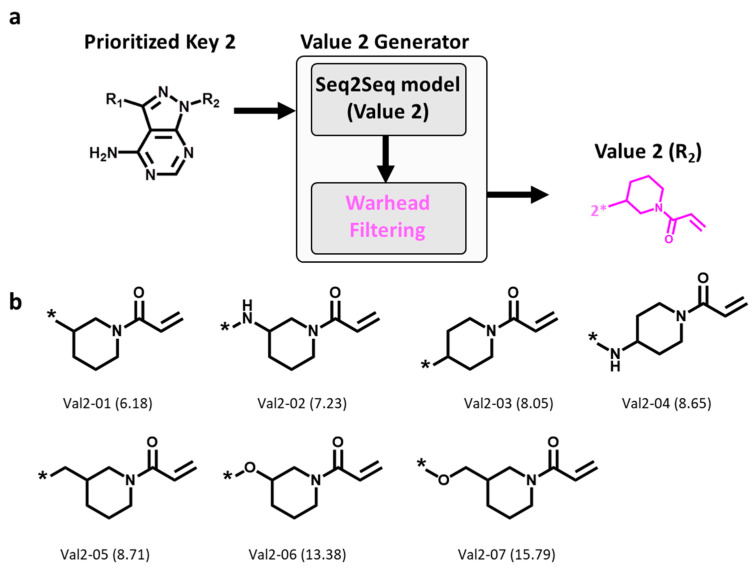
Generation of Value 2 fragments. (**a**) Workflow for generating Value 2 fragments using the Seq2Seq model (Value 2) and warhead filtering, and (**b**) new Value 2 fragments. Below each structure, the identification number and log-likelihood score (in parentheses) are provided. * indicates the fragment attachment point.

On the basis of the Key 2-01 prioritized by pharmacophore fitting, Value 2 fragments were generated and filtered for the presence of the invariant warhead, as illustrated in Figure 4a. A total of 10,000 Value 2 fragments were sampled, 7 of which were found to contain the warhead, as shown in Figure 4b. Thus, these findings confirmed the ability of DeepSARM modeling to reproduce the desired warhead. Moreover, similar to the observations made for Key 2 structures, these Value 2 fragments displayed modifications of the ring moiety attached to Michael acceptor group.

The selected Key 2 and Value 2 fragments were then combined to obtain Key 1 structures used as input for the generation of Value 1 fragments according to Supplementary Figure S2b. For each Key 1, 2000 Value 1 fragments were sampled and the top 100 Value 1 fragments with the lowest log-likelihood score (similar to known inhibitors) were selected for the generation of candidate compounds according to Supplementary Figure S2c. Value 1 fragments generated from [Key 2-01 − Value 2] fragments are shown in Supplementary Figure S3. Since Value 1 fragments represent substituents in newly assembled candidate compounds, preference was given here to fragments similar to those in known BTK inhibitors.

#### 2.4.3. Candidate Compounds

**Figure 5:**
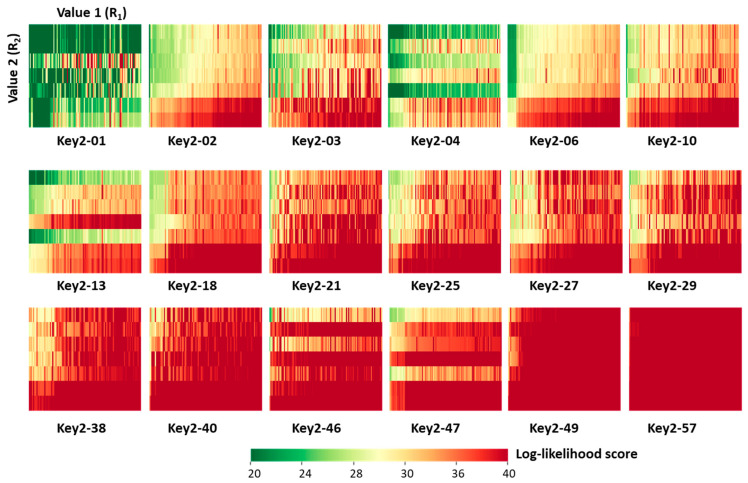
[Value 2 × Value 1] matrix for each of the 18 prioritized Key 2 fragments. The matrix cells represented unique ([Key 2 − Value 2] − Value 1) combinations (candidate compounds) color-coded by cumulative log-likelihood scores.

Next, we characterized the generated candidate inhibitors. For each of the 18 prioritized Key 2 structures, a 7 × 100 [Value 2 × Value 1] SARM-like matrix was generated in which matrix cells represented unique ([Key 2 − Value 2] − Value 1) combinations (candidate compounds) color-coded by cumulative DeepSARM log-likelihood scores, as shown in Figure 5. From the top left to the bottom right in Figure 5, matrices are arranged in the order of increasing scores, indicating increasing structural novelty compared to compounds used for fine-tuning (vide supra). As can be seen, for prioritized Key 2 fragments, compounds with varying structural novelty were obtained—an interesting finding.

**Figure 6:**
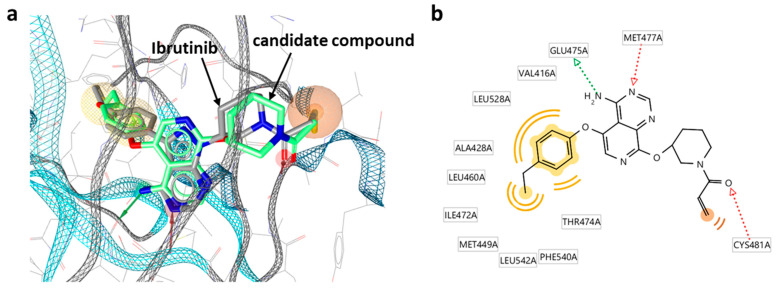
Hypothetical complexes of candidate inhibitors from DeepSARM and BTK. (**a**) Superposition of a candidate inhibitor containing Key 2-21 onto the crystallographic binding mode of ibrutinib. Pharmacophore features including two hydrogen bond acceptors, one hydrogen bond donor, one residue bonding point, and two optional hydrophobic features are represented as red arrows, green arrow, and orange/yellow sphere, respectively. (**b**) A corresponding diagram of candidate inhibitor–BTK interactions is shown.

The candidate inhibitors were then subjected to pharmacophore fitting using a compound-based pharmacophore model (see the Section 3) to prioritize compounds for follow-up analysis. Supplementary Figure S4 shows a Key 2-based matrix representation according to Figure 5 color-coded by pharmacophore score. With the exception of compounds containing Key 2-10, all matrices revealed small subsets of candidate compounds closely fitting the ibrutinib-BTK pharmacophore, while 34 different inhibitors were used for fine-tuning. Importantly, the number of compounds passing the pharmacophore filter did not inversely correlate with the structural novelty of the fragments forming the candidate compounds. For example, compounds containing Key 2-57 displayed the overall highest structural novelty but were also among the Key 2-based compound subsets most frequently matching the pharmacophore. Figure 6 shows a superposition of a candidate inhibitor containing Key 2-21 passing the pharmacophore filter onto the crystallographic binding mode of ibrutinib, and Supplementary Figure S5 shows examples of hypothetical complexes of BTK with candidate inhibitors obtained by pharmacophore fitting, indicating plausible binding modes.

These three compounds are representative candidates for further consideration. In all three cases, the acrylamide warhead is closely aligned with the targeted Cys481 residue (the carbonyl oxygen of the acrylamide warhead is positioned in hydrogen bonding distance to the thiol group). In addition, the differently substituted phenyl moieties in these compounds closely fit into a hydrophobic pocket in the active site of BTK distant from the reactive group, which further stabilizes binding. Importantly, the three candidate inhibitors contain different Key 2 structures, including two bicyclic cores (Key 2-21, with two fused six-membered rings; Key 2-49, fused six- and five-membered rings) and a tricyclic core (Key 2-04). Despite these chemically significant differences, these core fragments in these compounds are similarly positioned, including the tricyclic core, and interact with the same BTK residues (Glu475 and Met477). Thus, the putative binding modes closely resemble the experimental structure of ibrutinib and are plausible. The comparison of these candidate compounds suggests that there is a variety of opportunities for further chemical optimization.

Finally, the set of 1491 candidate compounds from DeepSARM passing the ibrutinib-BTK pharmacophore filter was compared to the 106 unique compounds with the piperidine-based Michael acceptor warhead contained in the high-confidence activity data subset of ChEMBL (Table 1). Only seven candidate compounds were contained in ChEMBL, revealing that the vast majority of BTK inhibitor candidates from DeepSARM represented new compounds. Corroborating insights were obtained by principal component analysis (PCA) of kinome inhibitor chemical space including DeepSARM candidate inhibitors (see the Section 3). As shown in Supplementary Figure S6, most of the known covalent BTK inhibitors containing the piperidine-based Michael acceptor warhead mapped to a peripheral region of kinome inhibitor space, while other BTK inhibitors were widely distributed over this space. Newly designed candidate compounds predominantly populated the region outlined by covalent BTK inhibitors used for fine-tuning, hence reflecting the desired focusing effect, and also further extended the kinome inhibitor space in this region with many new candidate compounds. Both focusing on known active compounds and generating chemical novelties around them were central aspects of the inhibitor design strategy reported herein.

## 3. Materials and Methods

### 3.1. DeepSARM Training

For pre-training of Seq2Seq models for Key 2, Value 2, and Value 1 generation, the number of epochs was set to 30, 10, and 30, respectively. For fine-tuning of Seq2Seq models for Key 2, Value 2, and Value 1, epochs were set to 50, 500, and 500, respectively. For all 3 models, the batch size was set to 64 and compound datasets were divided into training and validation sets (9:1) for pre-training and fine-tuning. Scripts for model derivation were written in Python and the Seq2Seq models were built using keras [16] (with 256-dimensional latent LSTM encoding space). Details of the DeepSARM architecture are provided as Supplementary Methods.

### 3.2. Fragment Generation Using DeepSARM

The Seq2Seq model (Key 2) was used to generate the Key 2 fragment, and the SMILES string [17] representing the ibrutinib Key 2 (“Nc1ncnc2c1c([At])nn2[*:1]”) (Figure 2a) was used as the input Key 2 fragment (where [At] and [*:1] are designated attachment points of Value 1 and Value 2, respectively). For sampling of 50,000 Key 2 fragments, the temperature factor was set to 2.0. Key 2 fragments without rotational bonds were selected. Value 2 fragments were generated from the Seq2Seq model (Value 2) using the ibrutinib Key 2 as the input fragment. For sampling of 10,000 Value 2 fragments, the temperature factor was set to 2.0. Value 2 fragments found to contain the piperidine-based Michael acceptor warhead were selected. Value 1 fragments were then generated with the Seq2Seq model (Value 1) using Key 1 fragments (assembled from Key 2 and Value 2 fragments) as the input. For each Key 1 fragment, 2000 Value 1 fragments were sampled, setting the temperature factor to 1.5.

### 3.3. Pharmacophore Modeling

For Key 2 fragment and candidate compound selection, two pharmacophore models were constructed from the co-crystal structure of ibrutinib bound to BTK (PDB [18] ID: 5p9j) using LigandScout 4.4 [19]. Both pharmacophore models were derived using an ensemble of exclusion volume spheres calculated based upon the X-ray structure of the ibrutinib-BTK complex.

To construct a pharmacophore model for Key 2 selection, three pharmacophore features were defined for the ibrutinib Key 2 fragments: aromatic, hydrogen bond acceptor, and hydrogen bond donor. In the X-ray structure, two hydrogen bonds were formed between the ibrutinib Key 2 and the hinge region of BTK (involving residues Glu475 and Met477). The pharmacophore model is shown in Figure 3a. The ‘idbgen’ module of LigandScout 4.4 was used for conformer generation of the 59 Key 2 fragments from the Seq2Seq model (Key 2). After conformer generation with default parameter settings, pharmacophore fitting was carried out setting the LigandScout scoring function to ‘Relative Pharmacophore-Fit’ and the conformation match mode to ‘BEST’.

For the selection of candidate compounds, a pharmacophore model with six pharmacophore features was derived, including two hydrogen bond acceptors, one hydrogen bond donor, one residue bonding point, and two optional hydrophobic features. The pharmacophore model is shown in Figure 6a. The residue bonding point feature is located in the vicinity of Cys481, which reacts with the warheads of covalent BTK inhibitors. After conformer generation using the ‘idbgen’ module with ‘icon-best’ parameter settings, pharmacophore fitting was carried out using ‘Relative Pharmacophore-Fit’ and setting the conformation match mode to ‘BEST’.

### 3.4. Principal Component Analysis

The 1491 DeepSARM candidate compounds were combined with the high-confidence kinase inhibitor data subset from ChEMBL and subjected to PCA. A total of 56,288 kinase inhibitors were compared to DeepSARM candidates, including 34 BTK covalent inhibitors, 929 other BTK inhibitors, and 55,325 inhibitors of other human kinases. For PCA, compounds were represented using extended-connectivity fingerprints [20] with bond diameter six (ECFP6) hashed to 2048-bit vectors. The first two principal components were used for generating a PCA plot.

## 4. Conclusions

In this work, we have introduced a computational approach for the design of covalent kinase inhibitors that combines fragment- and structure-based design components with deep generative modeling learning. As an exemplary application, the design of covalent BTK inhibitors containing an invariant piperidine-based acrylamide warhead was presented. Only limited information about specifically active known compounds was sufficient to effectively guide the design, reproduce a desired chemical warhead, as well as characteristic inhibitor substructures, and generate many novel candidate compounds. On the basis of the X-ray structure of the ibrutinib-BTK complex, candidate inhibitors were found to display meaningful chemical features and plausible binding modes. As demonstrated herein, the fragment-based design component of DeepSARM is well-suited for retaining chemical groups essential for covalent inhibition and embedding them into different structural environments inferred by deep learning from structures of kinase inhibitors. As presented in our proof-of-concept study, the approach for covalent inhibitor design is easily applicable to other targets and chemical warheads. For BTK, the exemplary kinase target investigated herein, nearly 1500 candidate inhibitors were obtained meeting the design constraints. As a part of our study, this set of candidate compounds (and the 34 BTK inhibitors used for fine-tuning) has been made freely available as an open-access deposition on the Zenodo platform [21] as a resource for medicinal chemistry applications on BTK and other TEC kinases.

## Figures and Tables

**Table 1:** Reported are inhibitors from ChEMBL containing the piperidine-based Michael acceptor warhead with activity against different kinases and their overlap with BTK inhibitors. Kinases with a free cysteine residue at a position corresponding to BTK are given in bold. # stands for Number.

Protein Kinase	# of Inhibitors with Warhead	# of BTK Inhibitors with Warhead
**Epidermal growth factor receptor erbB1**	35	1
**Tyrosine-protein kinase BTK**	34	34
Tyrosine-protein kinase JAK1	9	5
**Tyrosine-protein kinase JAK3**	8	6
Tyrosine-protein kinase JAK2	7	4
**Receptor protein-tyrosine kinase erbB-4**	4	3
**Tyrosine-protein kinase ITK/TSK**	4	2
Tyrosine-protein kinase TYK2	4	4
**Receptor protein-tyrosine kinase erbB-2**	3	2
**Tyrosine-protein kinase BLK**	3	3
**Tyrosine-protein kinase BMX**	3	2
**Tyrosine-protein kinase TEC**	2	1
Fibroblast growth factor receptor 1	2	0
Fibroblast growth factor receptor 2	2	1
**Tyrosine-protein kinase TXK**	2	2
Tyrosine-protein kinase receptor RET	1	1
Dual specificity mitogen-activated protein kinase kinase 1	1	1
Tyrosine-protein kinase SRC	1	0
Tyrosine-protein kinase Lyn	1	1
Tyrosine-protein kinase LCK	1	1

**Table 2:** Reported are the numbers of cyclic Key 2 substructures from newly designed BTK inhibitors detected in different sets of ChEMBL compounds with high-confidence activity data. Key 2 fragments not detected in any ChEMBL compounds are shown in bold. # stands for Number.

Key 2 Fragments	# of Covalent BTK Inhibitors	# of All BTK Inhibitors	# of Kinase Inhibitors	# of All ChEMBL Compounds
Key2-01	24	110	1021	2463
Key2-02	1	10	33	97
Key2-03	1	63	2239	2799
Key2-04	0	0	1	1
**Key2-06**	0	0	0	0
**Key2-10**	0	0	0	0
**Key2-13**	0	0	0	0
Key2-18	0	0	0	82
Key2-21	0	0	227	342
**Key2-25**	0	0	0	0
Key2-27	0	0	153	769
Key2-29	0	0	25	471
Key2-38	0	0	0	81
Key2-40	0	0	3	61
Key2-46	24	110	1021	2463
**Key2-47**	0	0	0	0
Key2-49	0	0	2	76
**Key2-57**	0	0	0	0
**Σ inhibitors in datasets**	**34**	**963**	**56,288**	**272,896**

## Data Availability

Newly designed BTK candidate inhibitors reported in this study are available as an open-access deposition via the following link: https://doi.org/10.5281/zenodo.5848494 (accessed on 14 January 2022).

## Supplementary Materials

The following supporting information can be downloaded online. Figure S1: Covalent BTK inhibitors with an acrylamide warhead; Figure S2: SAR matrix generation using DeepSARM; Figure S3: Value 1 fragments from DeepSARM for BTK inhibitor design; Figure S4: Key 2-based Value 1 × Value 2 matrices color-coded on the basis of pharmacophore scores; Figure S5: Hypothetical complexes of candidate inhibitors from DeepSARM with BTK; Figure S6: Principal component analysis of kinome inhibitor space. Supplementary Methods.
