# Supplementary material for: Approach for the Design of Covalent Protein Kinase Inhibitors via Focused Deep Generative Modeling
Source: Molecules. 2022 Jan 17;27(2):570. doi: 10.3390/molecules27020570 (PMC8778003; doi:10.3390/molecules27020570)
Supplement: Supplementary file 1 [file molecules-27-00570-s001.zip › molecules-1543026-supplementary.pdf]

Supplementary Materials

Approach for the Design of Covalent Protein Kinase Inhibitors via Focused Deep Generative Modeling

Atsushi Yoshimori [1,†], Filip Miljković [2,†], Jürgen Bajorath [2,*]

[1] Institute for Theoretical Medicine, Inc., 26-1 Muraoka-Higashi 2-chome, Fujisawa, Kanagawa 251-0012, Japan.

[2] Department of Life Science Informatics and Data Science, B-IT, LIMES Program Unit Chemical Biology and Medicinal Chemistry, Rheinische Friedrich-Wilhelms-Universität, Friedrich-Hirzebruch-Allee 6, D-53115 Bonn, Germany;

* Correspondence: bajorath@bit.uni-bonn.de; Tel.: +49-228-7369-100.

† These authors contributed equally.

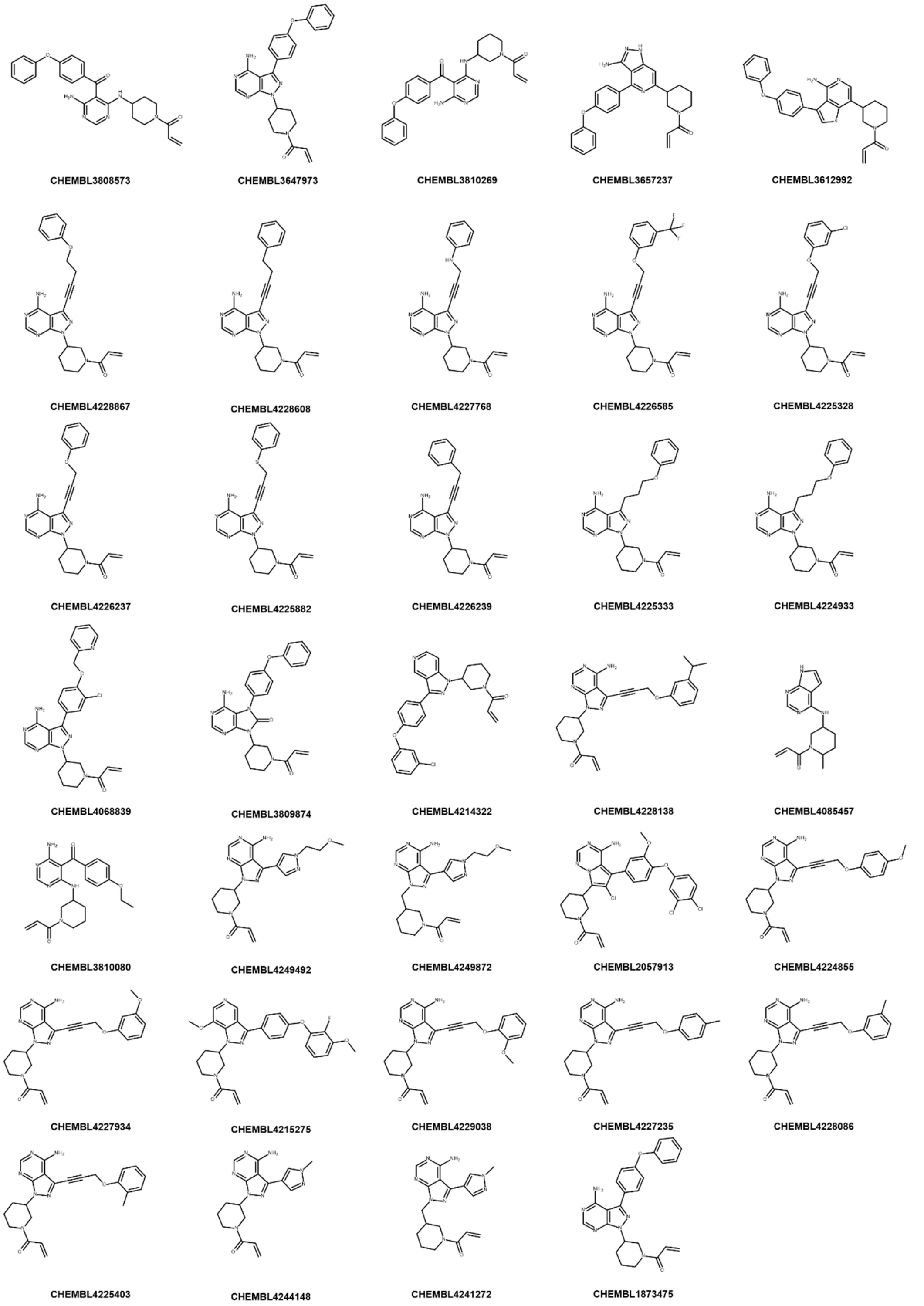

Supplementary Figure S1. Covalent BTK inhibitors with an acrylamide warhead. The 34 inhibitors shown here were used for fine-tuning the DeepSARM model. For each inhibitor, ChEMBL identifier is provided.

Supplementary Methods

DeepSARM

SAR Matrix concept

SAR matrices (SARMs) were originally designed to systematically extract analogue series (ASs) from large compound collections, organize structurally related ASs in matrices reminiscent of R-group tables, visualize SARs, and generate virtual candidate compounds to further expand ASs. The identification and organization of structurally related ASs is facilitated by a dual-step compound decomposition scheme [S1] akin to fragmentation of bonds for the generation of matched molecular pairs (MMPs) [S2]. In the first step, exocyclic single bonds in compounds are systematically cleaved applying size limitations for the resulting fragments, which yields keys (core structures, scaffolds) and values (substituents, R-groups) that are stored in an index table. This procedure identifies all analogues sharing a particular core with R-group replacements at a single site, hence defining a matching molecular series (MMS) [S3] for each structurally unique core. In the second step, all keys obtained in the first round are re-submitted to fragmentation, which then identifies all structurally analogous cores with a chemical change at a single site and the corresponding MMSs. Each subset of MMSs with unique structurally related cores is organized in an individual SARM such that each row contains an MMS and each column compounds from different series sharing the same R-group, as illustrated in **Supplementary Figure S2a**. Depending on the ASs contained in a given compound collection and their structural relationships, varying numbers of SARMs will be obtained. Each cell in a SARM represents a unique compound. SAR information is visualized by coloring cells according to compound potency. Hence, structural relationships and associated activity patterns can be traced within SARMs. Empty cells represent virtual analogs consisting of non-existing key-value (core-substituent) combinations, as also illustrated in **Supplementary Figure S2a.** Accordingly, virtual candidate compounds from SARMs further extend chemical space of related ASs and can be envisioned to form an envelope in chemical space around these series. The SARM methodology and resulting SARM data structure bridge between structure-activity relationship (SAR) visualization and compound design.

Deep learning extension

DeepSARM was based on the idea to further expand analogue design by taking information from compounds with activity against targets into account that are related to the primary target of interest [S4]. For example, a DeepSARM model can initially be trained with compounds active against the target family to which the target of interest belongs, followed by fine-tuning of the model for the primary target. This procedure increases the close-in analogue design capacity of the SARM approach. SARMs resulting from original two-step fragmentation can then be further expanded with novel key and value fragments and additional SARMs entirely consisting of novel fragments and compounds can be obtained.

For generative design on the basis of key and value fragments encoded as canonical SMILES strings [S5], an encoder-decoder framework consisting of long short-term memory (LSTM) units [S6] represents a preferred recurrent neural network (RNN) architecture [S7]. The encoder-decoder framework is used to derive sequence-to-sequence (Seq2Seq) models that translate one sequence of one-hot encoded SMILES strings into another [S8]. The encoder LSTM transforms input sequences into two-state vectors in latent space and the decoder LSTM is trained to return the same sequences as transformed SMILES. For DeepSARM, encoder-decoder units were built using keras [S9] (with 256-dimensional latent LSTM encoding space).

DeepSARM includes three encoder-decoder units for the generation of Seq2Seq models, as illustrated in **Supplementary Figure S2b**. The Seq2Seq model for key 2 (i.e., the key 2 generator) is trained using input key 2 / output key 2 pairs, the model for value 2 using key 2 / value 2 pairs, and the model for value 1 using key 1 / value 1 pairs. Compounds with newly generated key fragments are added to an original SARM containing structurally analogous keys (meeting the step-2 fragmentation criterion) and hence further expand the SARM.

The three Seq2Seq models are derived as follows:

(I) Model (key 2) using key 2 (input) / key 2 (target) pairs;

(II) Model (value 2) using key 2 (input) / value 2 (target) pairs;

(III) Model (value 1) using key 1 (input) / value 1 (target) pairs.

Pre-training is carried out using large numbers of compounds with activity against a target family or set and fine-tuning using a comparably smaller set of compounds active against the primary target. During fine-tuning, internal model weights are adjusted.

The Seq2Seq models generate key 2, value 2, and value 1 fragments that are evaluated on the basis of a log-likelihood score:

$$\log - likelihood\ score\ = -\sum_{t=1}^{T} logP(x^t|x^{t-1}, \dots, x^1)$$

P represents the probability distribution of the decoder and *T* the number of SMILES tokens for a given fragment. The minus sign ensures that high probabilities result in small scores for fragment prioritization.

A log-likelihood threshold can be applied to filter fragments. Compounds are then obtained by combining newly generated key 1 and value 1 fragments. Further DeepSARM calculation details are provided in [S4].

Supplementary Figure S2c illustrates the generation of a SARM using the Seq2Seq models for value 2 and value 1. Key 2 fragments provide the input for the Seq2Seq model generating value 2 fragments. Key 1 is then constructed from key 2 and value 2 fragments. The resulting key 1 fragments serve as is input for the Seq2Seq model of value 1. New compounds comprising the SARM are then obtained by combining the resulting key 1 and value 1 fragments. Each cell of the SARM represents a new compound and is color-coded by the combined log-likelihood score. Invalid SMILES strings are removed and value 1 and value 2 filters are applied as SMARTS screens to remove chemically undesired substituents (such as unstable fragments).

The DeepSARM description above and **Supplementary Figure S2** were adopted from open access reference [S10].

Supplementary References

S1. Wassermann, A. M.; Haebel, P.; Weskamp, N.; Bajorath, J. SAR Matrices: Automated Extraction of Information-Rich SAR Tables from Large Compound Data Sets. *J. Chem. Inf. Model.* **2012**, *52*, 1769-1776.

S2. Hussain, J.; Rea, C. Computationally Efficient Algorithm to Identify Matched Molecular Pairs (MMPs) in Large Data Sets. *J. Chem. Inf. Model.* **2010**, *50*, 339-348.

S3. Wawer, M.; Bajorath, J. Local Structural Changes, Global Data Views: Graphical Substructure-Activity Relationship Trailing. *J. Med. Chem.* **2011**, *54*, 2944-2951.

S4. Yoshimori, A.; Bajorath, J. Deep SAR Matrix: SAR Matrix Expansion for Advanced Analog Design Using Deep Learning Architectures. *Future Drug Discov.* **2020**, *2*, FDD36.

S5. Weininger D. SMILES, a Chemical Language and Information System. 1. Introduction to Methodology and Encoding Rules. *J. Chem. Inf. Comput. Sci.* **1988**, *28*, 31–36.

S6. Hochreiter, S.; Schmidhuber, J. Long Short-Term Memory. *Neur. Comput.* **1997**, *9*, 1735–1780.

S7. Zheng, S.; Yan, X.; Gu, Q.; Yang, Y.; Du, Y.; Lu, Y.; Xu, J. QBMG: Quasi-Biogenic Molecule Generator with Deep Recurrent Neural Network. *J. Cheminf.* **2019**, *11*, e5.

S8. Sutskever, I.; Vinyals, O.; Le, Q. V. Sequence to Sequence Learning with Neural Networks. *Adv. Neur. Inf. Proc. Sys.* **2014**, *1*, 3104–3112.

S9. Ketkar, N. Introduction to keras. In: Deep learning with Python. Apress, Berkeley, CA, 97-111, 2017.

S10. Yoshimori, A.; Bajorath, J. Iterative DeepSARM Modeling for Compound Optimization. *Artif. Intell. Life Sci.* **2021**, *1*, e100015.

(a)

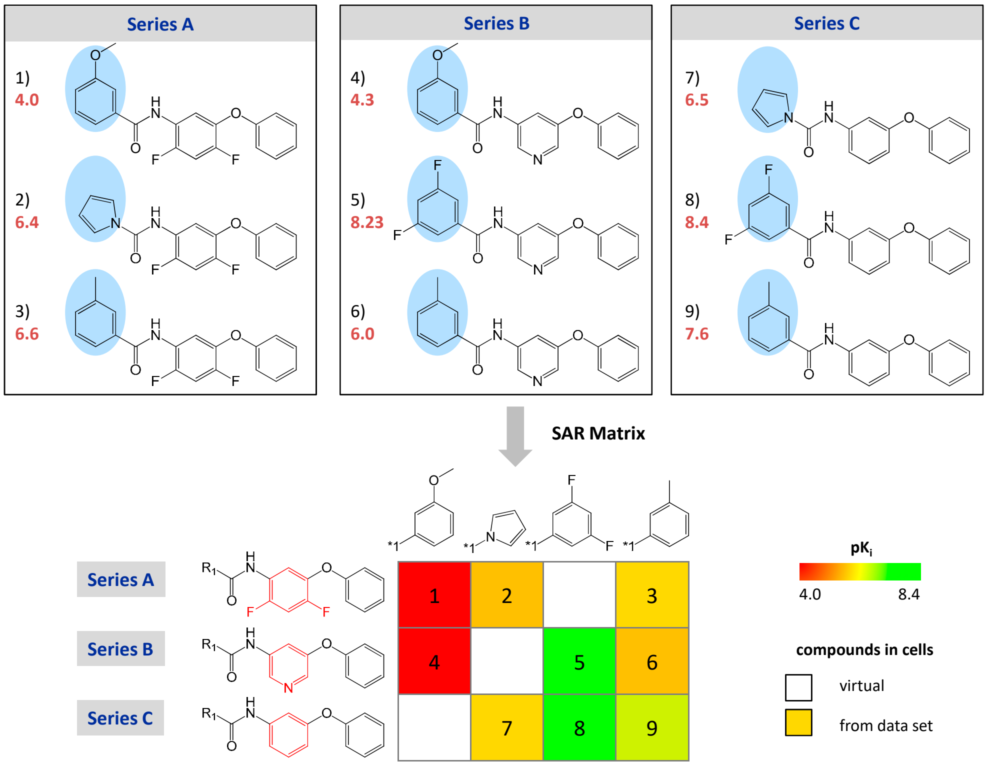
Series A
1)
4.0
2)
6.4
3)
6.6
Series B
4)
4.3
5)
8.23
6)
6.0
Series C
7)
6.5
8)
8.4
9)
7.6
SAR Matrix
Series A
Series B
Series C
1
2
3
4
5
6
7
8
9
pKi
4.0
8.4
compounds in cells
virtual
from data set

(b)

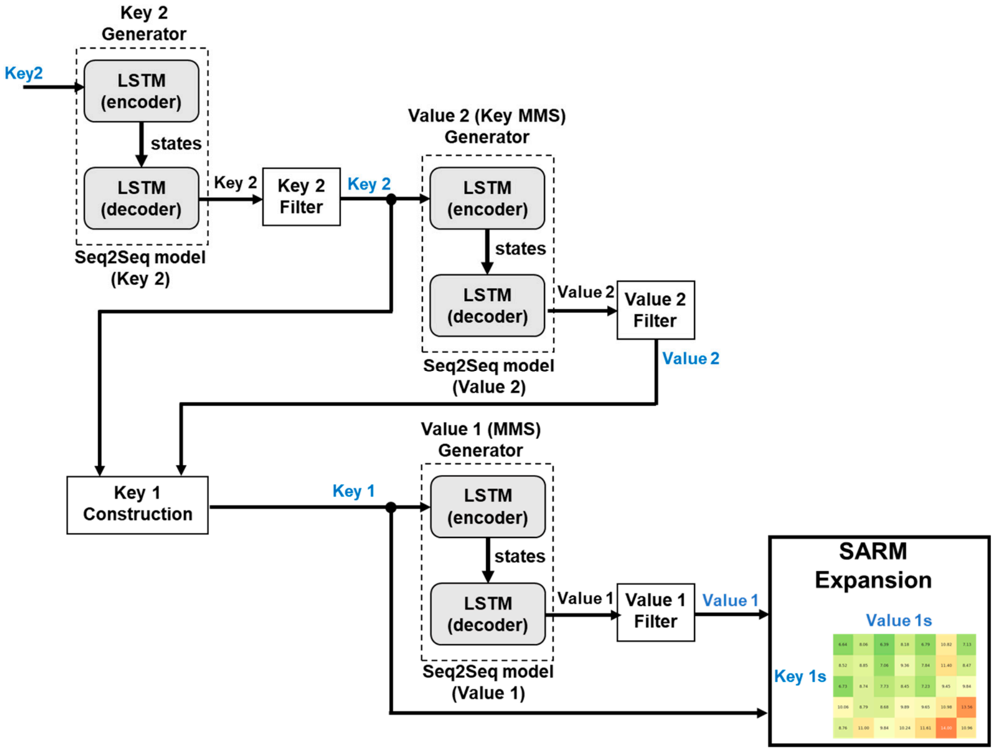
Key 2 Generator
Key2
LSTM (encoder)
states
LSTM (decoder)
Seq2Seq model (Key 2)
Key 2
Key 2 Filter
Key 2
Value 2 (Key MMS) Generator
LSTM (encoder)
states
LSTM (decoder)
Seq2Seq model (Value 2)
Value 2
Value 2 Filter
Value 2
Value 1 (MMS) Generator
Key 1 Construction
Key 1
LSTM (encoder)
states
LSTM (decoder)
Seq2Seq model (Value 1)
Value 1
Value 1 Filter
Value 1
SARM Expansion
Value 1s
Key 1s

(**c**)

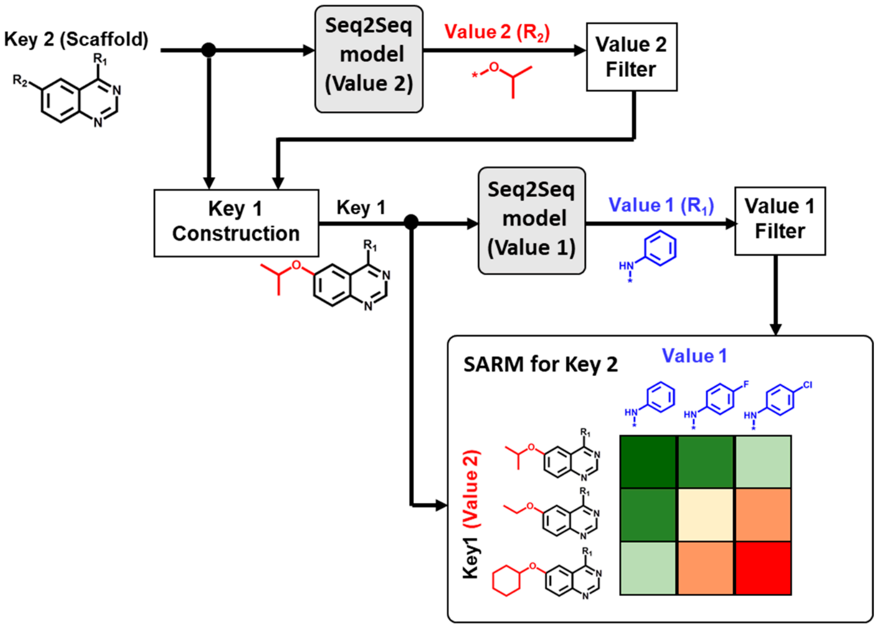

Supplementary Figure S2. SAR matrix generation using DeepSARM. (**a**) SARM design is illustrated using three small structurally related compound series (A, B, and C). Analogues from the different series are consecutively numbered and their pK_i values are reported in red. Distinguishing substituents are shown on a blue background and substructures differentiating scaffolds (keys) are colored red. In the SARM, each row contains an MMS and each column compounds from different series with the same substituent (values). Existing analogs are represented by cells that are color-coded by activity. In addition, empty cells represent virtual analogues. (**b**) The architecture of DeepSARM is illustrated, which consists of three LSTM of encoder-decoder units. (**c**) The construction of a SARM with DeepSARM is illustrated using Seq2Seq models for value 2 and value 1. Key 2 is a scaffold, which represents the input of the Seq2Seq model for generating value 2, the R_2 substituent of the key 2 scaffold. Key 1 is constructed from key 2 and value 2. Key 1 is the input of the Seq2Seq model for value 1, the R_1 substituent of key 1 or key 2. The SARM is constructed from the resulting key 1 and value 1 fragments and color-coded by log-likelihood scores. Value 1 and value 2 filters represent structural screens to remove chemically questionable substituents.

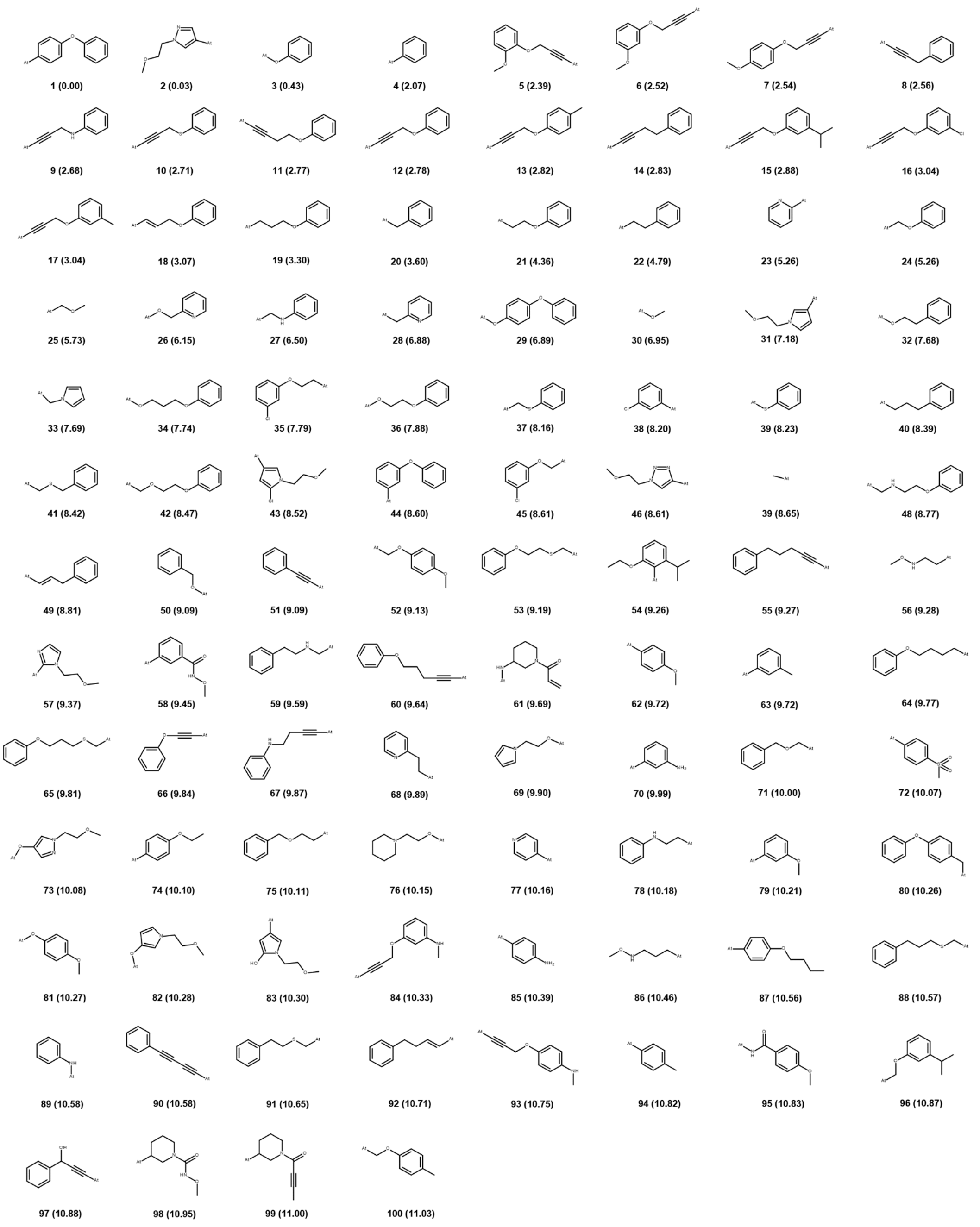

Supplementary Figure S3. Value 1 fragments from DeepSARM for BTK inhibitor design. Shown are Value 1 fragments generated from [Key 2-01–Value 2] fragments. Value 1 identification numbers and log-likelihood scores (in parentheses) are reported below each structure.

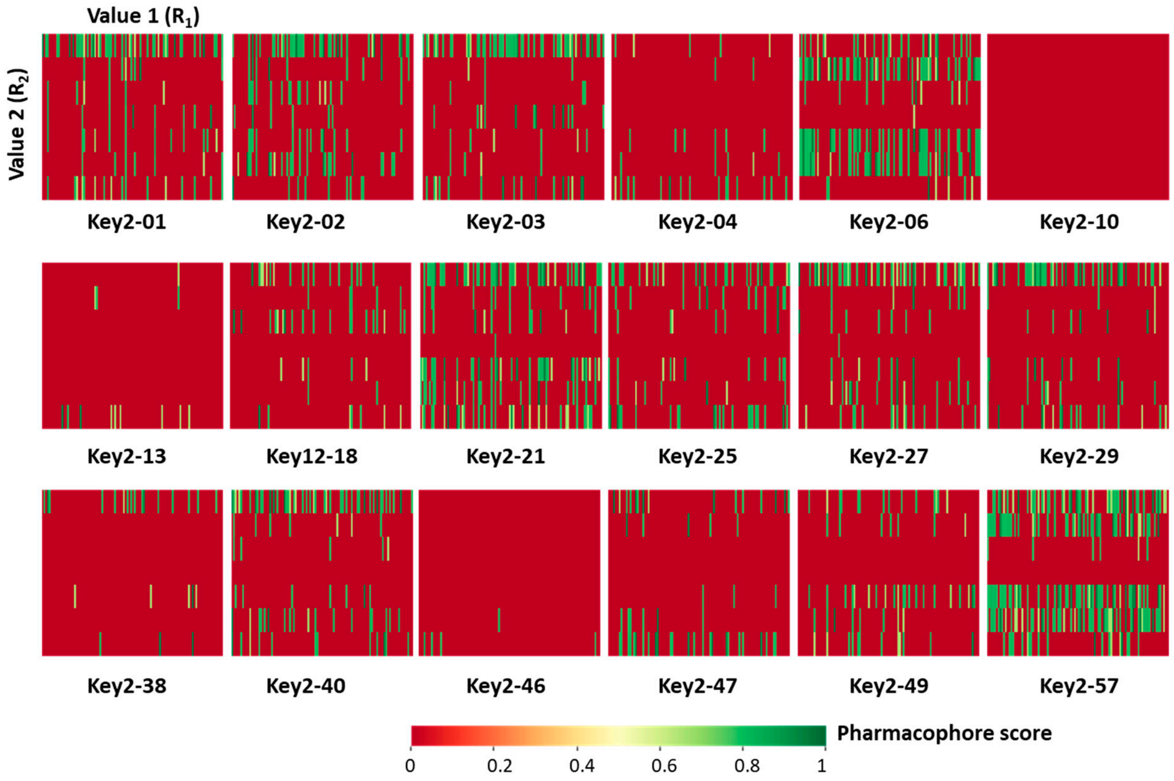

Supplementary Figure S4. Key 2-based Value 1 x Value 2 matrices color-coded on the basis of pharmacophore scores. Each matrix cell represents a unique ([Key 2–Value 2]–Value 1) combination (candidate compound).

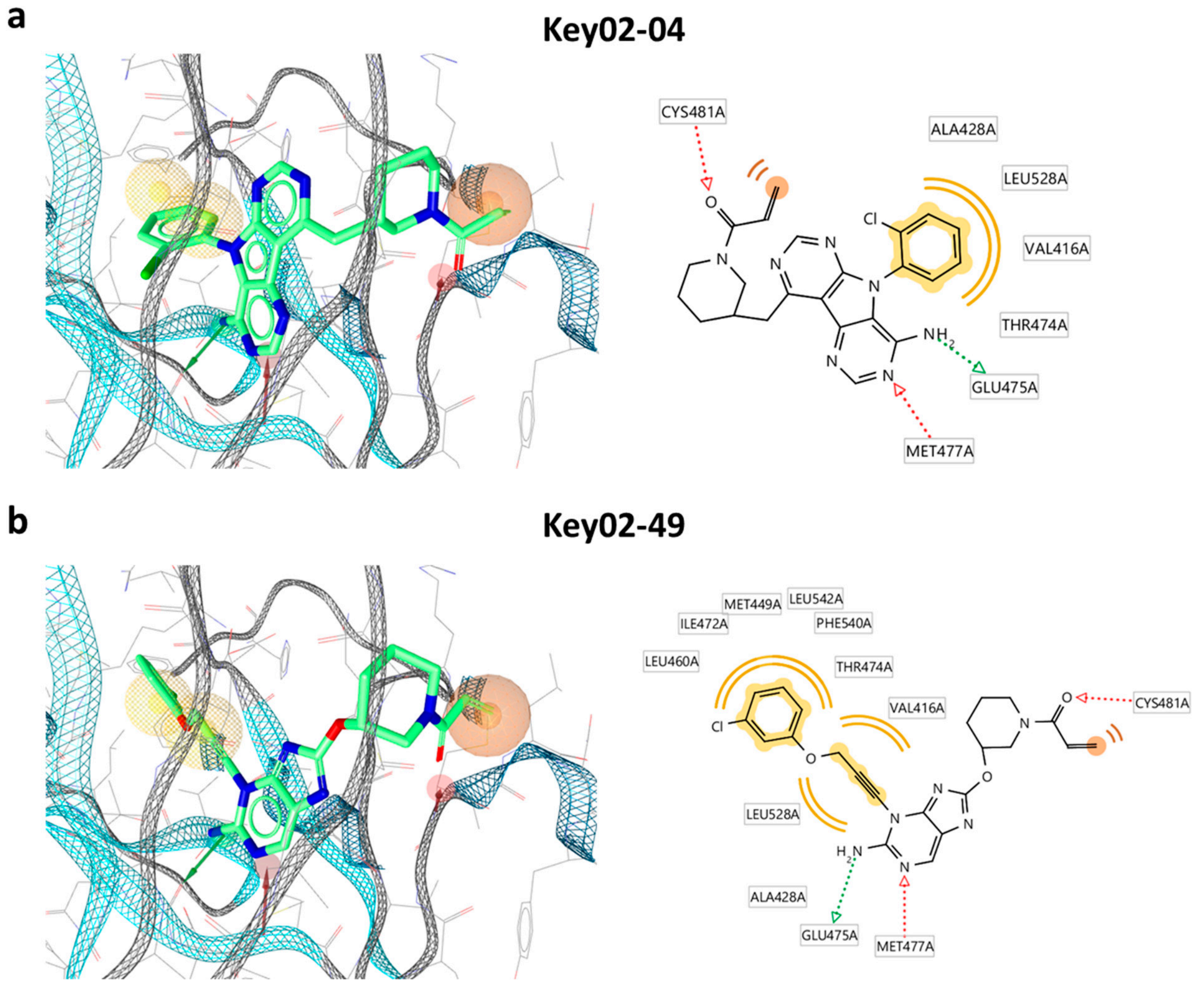

Supplementary Figure S5. Hypothetical complexes of candidate inhibitors from DeepSARM with BTK. In (**a**) and (**b**), two exemplary models of complexes are shown (left) together with corresponding ligand-kinase interaction diagrams (right). The covalent bond to the thiol group free cysteine (orange sphere) is not drawn.

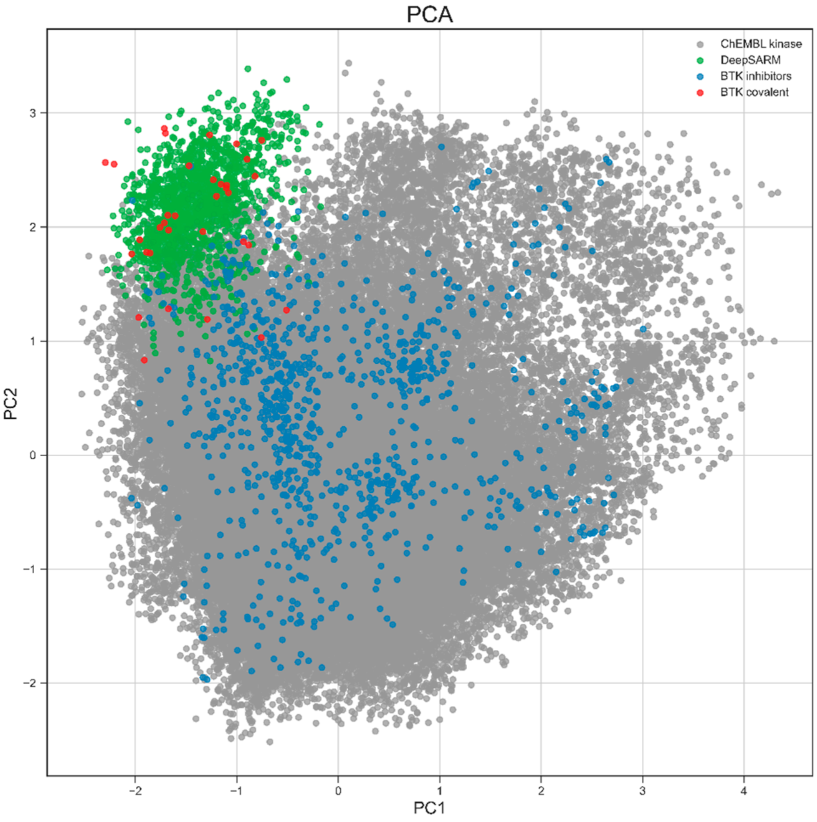

Supplementary Figure S6. Principal component analysis of kinome inhibitor space. Shown is a PCA plot of a combined set of DeepSARM candidate compounds (green), covalent BTK inhibitors used for fine-tuning (red), other BTK inhibitors (blue), and inhibitors of other human kinases (gray).
